# MAPK Signaling in the Interplay Between Oxidative Stress and Autophagy

**DOI:** 10.3390/antiox14060662

**Published:** 2025-05-30

**Authors:** Enrico Desideri, Serena Castelli, Maria Rosa Ciriolo

**Affiliations:** 1 Department of Life Sciences, Health and Health Professions, Link Campus University, Via del Casale di San Pio V, 44, 00165 Rome, Italy; e.desideri@unilink.it; 2 Department of Human Sciences and Promotion of the Quality of Life, San Raffaele Roma Open University, Via di Val Cannuta, 247, 00166 Rome, Italy; serena.castelli@uniroma5.it; 3 IRCCS San Raffaele Roma, Via di Val Cannuta, 247, 00166 Rome, Italy; 4 Department of Biology, University of Rome “Tor Vergata”, Via Della Ricerca Scientifica, 00133 Rome, Italy

**Keywords:** autophagy, MAPK, oxidative stress, CMA, eMI, atypical MAPK

## Abstract

The term autophagy identifies several mechanisms that mediate the degradation of intracellular and extracellular components via the lysosomal pathway. Three main forms of autophagy exist, namely macroautophagy, chaperone-mediated autophagy, and endosomal microautophagy, which have distinct mechanisms but share lysosomes as the final destination of their cargo. A basal autophagic flux is crucial for the maintenance of cellular homeostasis, being involved in the physiological turnover of proteins and organelles. Several stressors, including nutrient shortage and genotoxic and oxidative stress, increase the autophagic rate, which prevents the accumulation of damaged and potentially harmful cell components, thus preserving cell viability. In this context, several studies have highlighted the role of MAPKs, serine–threonine kinases activated by several stimuli, in linking oxidative stress and autophagy. Indeed, several oxidative stressors activate autophagy by converging on MAPKs, directly or indirectly. In this regard, the different transcription factors that bridge MAPKs and autophagic activation are here described. In this review, we summarize the current knowledge regarding the regulation of autophagy by MAPK, including the atypical ones, with a particular focus on the regulation of autophagy by oxidative stress.

## 1. Introduction

Autophagy is a collective term that identifies different evolutionary conserved mechanisms involved in the elimination of intracellular and extracellular material via the lysosomes. It plays a pivotal role in maintaining cellular homeostasis, as it can degrade damaged or aggregated proteins, organelles, as well as intracellular pathogens, preventing negative consequences [1]. According to the specificity of substrates degraded by autophagy, autophagy can be divided into non-selective and selective autophagy; the latter refers to the degradation of specific organelles (e.g., mitophagy, ribophagy, pexophagy, and so on) or proteins. Selective autophagy usually requires adaptor proteins that recognize the protein/organelle to be degraded and direct the assembly of the autophagic machinery. Selective autophagy is important during cell differentiation, development, and aging [2]. While autophagy is always active at low rates in physiological conditions, its activity is boosted when cells face stress conditions, such as nutrient deprivation or oxidative stress. In this condition, autophagy acts as a survival mechanism that degrades non-essential or damaged molecules to limit cell damage and/or use their building blocks to sustain the fundamental functions of the cell [3].

In this review, we describe the current knowledge about the regulation of the different types of autophagy by mitogen-activated protein kinases (MAPKs), a family of kinases that responds to both intrinsic and extrinsic stimuli and acts as a key determinant of cell fate upon stress conditions, being involved in the regulation of both death and survival mechanisms. In particular, we will highlight how the different MAPKs influence autophagy in conditions of oxidative stress and the underlying molecular mechanism.

## 2. Mechanism and Functions of Autophagy

### 2.1. Mechanism of Autophagy

**Figure 1:**
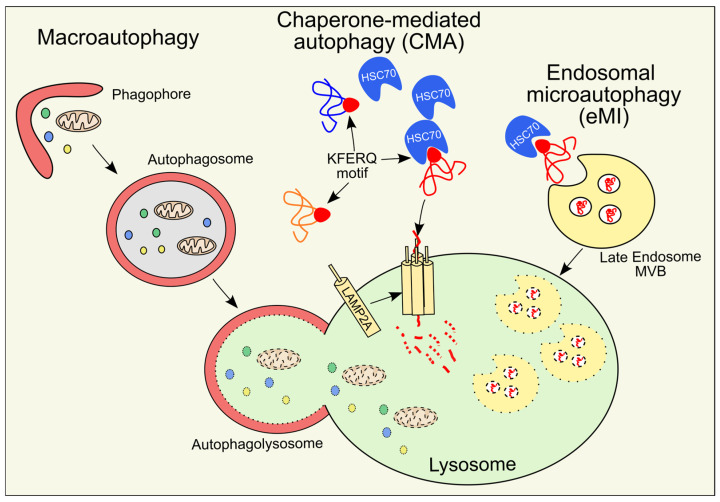
Mechanisms of the different types of autophagy, namely macroautophagy, chaperone-mediated autophagy (CMA), and endosomal microautophagy (eMI), all converging into lysosomal degradation.

Among the many variants of autophagy so far cited, the three main types are: macroautophagy; endosomal microautophagy (eMI); and chaperone-mediated autophagy (CMA) (Figure 1). They differ in the way the substrates are recognized and transported to the lysosomes for degradation [2]. In macroautophagy, portions of the cytoplasm or single organelles are engulfed into double-membraned structures, while in eMI and CMA, the cargo is captured by the endosomes and the lysosomes, respectively.

#### 2.1.1. Macroautophagy

In macroautophagy (hereinafter called autophagy) cytoplasmic cargoes are sequestered into double-walled membranes called autophagosomes, which originate from the expansion of crescent-shaped structures, the phagophore, and then fuse with the lysosomes to allow the degradation of their content. Thus, the process includes several steps, including phagophore formation, elongation, and autophagosome–lysosome fusion, each regulated by one or more regulatory complexes. The initiation of autophagosome formation is governed by the unc-51-like kinase (ULK) complex, a multiprotein complex composed of FIP200, ATG13, ATG101, and ULK1/ULK2 [3]. ATG13 forms a heterodimer with ATG101, representing a subcomplex binding ULK1 and FIP200, which contains the catalytic domain [4].

Under normal conditions, the ULK1 complex is ubiquitously localized in the cell and inhibited by the mammalian target of rapamycin complex 1 (mTORC1). After a stimulus that induces autophagy, mTORC1 is inactivated, allowing the activation of the ULK complex, which forms visible dots in proximity to the endoplasmic reticulum (ER) membrane, co-localizing specifically with omegasomes, ER structures supporting autophagosome formation [5]. After ULK1/ULK2 is activated, phosphorylation of ATG13 and FIP200 and autophosphorylation of ULK proteins occur. The active ATG13-ULK-FIP200 complex recruits other autophagy-related (ATG) proteins determining the initiation of autophagosome formation and culminating in nucleation, with the activation of class III phosphatidylinositol 3-kinase (PIK3C3) complex, composed of BECN1, AMBRA1, ATG14L, VPS15, and VPS34 and that produce phosphatidylinositol 3-phosphate (PIP3) on the ER membrane, leading to the recruitment of WD-repeat protein interacting with phosphoinositides (WIPI) proteins [4]. There are four WIPI proteins, but WIPI2 is the most abundant and its depletion profoundly impacts autophagy [5]. WIPI2 interacts with ATG16L1, included in the ATG5–ATG12–ATG16L1 (E3-like) complex, and recovers ATG9-positive vesicles allowing the autophagosome nucleation [4]. The E3 Complex promotes the lipidation of different ATG8 proteins, which include LC3 and GABARAP, allowing the binding of ATG2 through its LC3-interacting region (LIR). ATG2 is considered a lipid transfer unit from the ER to the de novo synthesized organelle by supplying phospholipids for autophagosome formation [6,7].

The lipidation of LC3 is a multi-step process similar to ubiquitination that starts with the cleavage of newly synthesized LC3 (pro-LC3) by ATG4 to form LC3-I, which localizes to the cytosol. LC3-I has a C-terminal glycine residue that is susceptible to conjugation. The conjugation requires the adenylation by the ATG7 (E1-like), forming a thioester intermediate. Then, LC3-I binds ATG3 (E2-like) and is finally conjugated with the amine group of phosphatidylethanolamine (PE) localized to the nascent autophagosomes. The amount of lipidated LC3 (LC3-II) is widely used as an indicator of the number of autophagosomes in the cells [8]. The presence of LC3-II on both the inner and outer membrane of autophagosomes allows not only autophagosome formation but also cargo selection via the interaction with several adaptor proteins harboring an LIR motif (e.g., p62, BNIP3) [9]. Recently, human ATG4 has been also described as capable of autophagosome formation independently of its protease activity and able to traffic ATG9-vesicles, with its proximity network. This non-canonical function is carried out specifically during PINK1/Parkin-dependent mitophagy. Indeed, the disruption of the four ATG4s genes using the CRISPRCas9 technique elegantly highlighted that ATG4A and ATG4D, together with proximal proteins involved in the transport process and lipid modification (including LPS responsive beige-like anchor protein, LRBA), play a role in ATG9 recruitment during mitophagy [10]. ATG4 also contributes to LC3-II deconjugation, which allows the release and recycling of LC3. ATG4B has been proposed as the main responsible for LC3 de-lipidation. Its regulation occurs through ULK1-mediated phosphorylation on serine 316 of ATG4B, which inhibits its catalytic activity. On the contrary, phosphatase PP2A-PP2R3B can dephosphorylate ATG4B. These opposite activities of ULK1 and PP2A proteins regulate the cellular activity of ATG4B and, consequently, LC3 processing [9].

The specificity of the autophagic system is given by proteins known as autophagic receptors that facilitate the engulfment of specific cargoes into autophagosomes. The main feature of autophagic receptors is the presence of both a LIR sequence in their structure, which mediates the binding with LC3-II, and a ubiquitin-binding domain (UBD), which allows them to act as a bridge between cargo to be degraded and the forming autophagosome. In the majority of cases, autophagic receptors bind poly-ubiquitin chains, but they can also have a direct interaction with the cargo. In this latter case, the expression of cargo-localizing receptors is directly regulated by stress conditions, including starvation and hypoxia [11]. A crucial example of a cargo-localizing receptor is the nuclear receptor coactivator 4 (NCOA4), which has been recently identified as an autophagic receptor specifically recognizing ferritin heavy-chains (FTH1). Excessive intracellular iron is sequestered by ferritin, to prevent harmful consequences. In case of low iron levels, NCOA4 conveys ferritin-iron complexes into the lysosome, where ferritin is degraded, and free iron is released into the cytosol. This process is referred to as ferritinophagy [12,13,14].

In the case of autophagic receptors binding poly-ubiquitin chains, ubiquitination of substrates by ubiquitin ligases is the major regulatory mechanism. This process allows proteins or organelles that cannot be repaired to be specifically marked and represents a particular advantage in recognizing damaged organelles contained in a network, such as that of mitochondria and ER. In the former, autophagic degradation must be preceded by an isolation process. The selective degradation of mitochondria by autophagy (mitophagy) is preceded by mitochondria fragmentation (fission) due to the activity of dynamin-related protein 1 (DRP1), mitochondrial fission factor (MFF), and mitochondrial fission protein 1 (FIS1) proteins. On the contrary, the activity of proteins involved in mitochondrial fusion, such as mitofusin 1 and 2 (MFN1 and MFN2) is inhibited [15].

For ER (ER-phagy), the mechanism is widely unclear; FAM134B has been identified as a receptor involved in the selectiveness of autophagy but it is not the only player [16].

Virtually all organelles can be degraded by selective macroautophagy. In addition to the above-mentioned mitophagy and ER-phagy, also lipid droplets (lipophagy), protein aggregates (aggrephagy), lysosomes (lysophagy) and pathogens (xenophagy) can be degraded by autophagy [17].

The removal of ubiquitin from the substrate is mediated by deubiquitinating enzymes (DUBs), which cleave single or poly-ubiquitin chains from proteins, releasing free ubiquitin. The deubiquitination process impacts biological processes, such as DNA damage response and DNA repair pathways, and it is involved in the regulation of cancer metabolism [18,19].

The best-studied autophagy receptor is sequestosome-1 (SQSTM-1/p62) [20]. Ubiquitin chains are recognized and bound by p62 monomers with low affinity, but following the homo-oligomerization, p62 oligomers show high avidity to bind poly-ubiquitin chains [13]. The affinity between the ubiquitin-associated domain (UBA) of p62 and the cargoes to be degraded is regulated by S403 phosphorylation, which can stabilize the sequestosome structure. The S403 phosphorylation of the UBA domain of p62 is mediated by Casein kinase 2 (CK2). Interestingly, it has been demonstrated that CK2 over-expression reduces mutant Huntingtin aggregation, consistent with the knowledge that toxic protein aggregations are cleared through autophagy [21].

Other autophagic receptors binding poly-ubiquitin chains in mammalian cells are neighbors of BRCA1 (NBR1), Tax1 binding protein 1 (TAX1BP1), nuclear dot protein 52 (NDP52), and optineurin. Together with p62, they all are soluble proteins having domains capable of binding ubiquitin marks. Considering the similarity to p62, they are all defined as sequestosome-like cargo receptors (SLRs) [22].

Autophagosomes containing their cargo are transported to the lysosomes using dynein motor proteins. The mature autophagosomes acquire SNARE proteins, which are also present on the lysosome membrane. When these two organelles are close enough, SNARE proteins form trans-SNARE complexes, namely an α-helical bundle that forces the fusion of the lysosomal membrane with the autophagosome outer membrane. Two SNARE complexes are involved in autophagosome and lysosome membrane fusion, classified based on the presence of a conserved glutamine (Q) (Q-SNARE) or arginine (R) (R-SNARE) residue in the SNARE motif [23].

The organelle deriving from the fusion between autophagosome and lysosome is called the autophagolysosome. After the autophagosome fusion with the lysosome, the acidic lysosomal hydrolases initially break down the autophagosome inner membrane and finally the autophagosome cargo [24]. Lysosomes contain more than 60 hydrolases, which include proteases, sulfatases, nucleases, lipases, phosphatases, glycosidases, and nucleases. They allow the cells to degrade many types of cargo, including nucleic acids and bacteria. Among lysosomal hydrolases, cathepsins are the most abundant. They are proteases classified into serine cathepsins (A and G), aspartic cathepsins (D and E), and cysteine cathepsins.

These enzymes optimally work in the lysosomal acidic conditions (pH 4.5–5.5), maintained by an H^+^-ATPase, or V-ATPase, present at the lysosomal membrane and that translocates two protons into the lysosome by consuming one ATP. The requirement of an acidic pH prevents cell auto-digestion in case of lysosomal damage and the release of lysosomal enzymes into the cytosol.

When the degradation is complete, macromolecule building blocks are transported out of the lysosomes via exporters or vesicular trafficking to be used for ATP production or as precursors of anabolic pathways [25].

#### 2.1.2. Chaperone-Mediated Autophagy

Chaperone-mediated autophagy (CMA) is a selective type of autophagy that degrades proteins bearing a recognition motif (KFERQ-like motif), biochemically related to the KFERQ sequence present in the first CMA substrate identified, RNAse A. The KFERQ motif is recognized by the chaperone HSPA8/HSC70 (heat shock protein family A (Hsp70) member 8) [26]. The KFERQ-like motif is present in a good percentage (up to 30%) of cytosolic proteins and is sufficient to allow their degradation by CMA, but it is often not accessible to HSC70 unless the protein is at least partially unfolded and/or modified by PTM. The HSC70-protein complex promotes the interaction with LAMP2A, the CMA receptor located on the lysosome membrane. The interaction of the CMA substrate with the cytosolic tail of LAMP2A promotes LAMP2A oligomerization and the formation of a multiprotein complex that facilitates substrate unfolding and internalization. Once the LAMP2A complex completes this role, it returns to the monomeric form, ready for a new translocation cycle. This assembly/disassembly dynamic of LAMP2A is regulated by glial fibrillary acidic protein (GFAP) and elongation factor 1α (EF1α). GFAP is responsible for the stabilization of the LAMP2A multimeric complex, responsible for the internalization of the substrate. The phosphorylated form of GFAP, instead, has a higher affinity for EF1α, forming with it a complex on the lysosomal membrane. When the substrate is translocated, EF1α is released and pGFAP becomes available to be bound. The affinity of GFAP for pGFAP is higher than that for LAMP2A; for this reason, GFAP/pGFAP dimer forms and GFAP/LAMP2A complex disassembles [27].

Another regulatory mechanism of CMA is mTORC2, which negatively impacts CMA by phosphorylating and activating AKT. Active AKT, indeed, can phosphorylate GFAP releasing it from LAMP2A, disassembling the translocation complex of CMA. On the contrary, a positive regulation depends on the Pleckstrin homology (PH) domain and leucine-rich repeat protein phosphatase 1 (PHLPP1) since it can remove the phosphorylation of Akt induced by mTORC2. In the same way, Torin-1 inhibits the function of mTORC2, by blocking the activation of AKT and inducing CMA [26,27,28].

The regulation can also occur at the level of chaperones responsible for substrate translocation, such as HSPA8 and heat shock protein 90 (Hsp90). These chaperones bind LAMP2A, regulating its recycling [29].

The levels of LAMP2A are crucial in the regulation of the CMA pathway. The transcription factor NFAT-1 can regulate the expression of LAMP2A based on intracellular ROS levels. Indeed, an increase in ROS leads to the recruitment of NFAT-1 on the LAMP2 promoter, upregulating its expression. In light of this, modulation of ROS is used as a suitable approach to regulate CMA. For instance, 6-Aminonicotinamide (6-AN), a ROS inducer, promotes CMA, as well as, vitamin E, as an antioxidant, reduces CMA levels [30].

#### 2.1.3. Endosomal Microautophagy (eMI)

In endosomal microautophagy (eMI), the cytoplasmic cargo is engulfed by late endosome/multi-vesicular bodies. The initial step, shared with CMA, is the recognition of a pentapeptide amino acid KFERQ-like targeting motif in the substrate protein by the HSC70 chaperone. This chaperone, through interaction with the chaperone Bag6, interacts with phosphatidylserine residues of late endosomes, instead of lysosomes as it occurs in CMA. The internalization inside the vesicles is mediated by the endosomal sorting complexes required for transport (ESCRT) machinery (Tsg101 and Alix) and the ATPase Vps4, which is responsible for the invagination of the membrane [31]. In the formed multivesicular bodies, the KFERQ-containing protein can be degraded after fusion with lysosomes or secreted after the fusion with the plasma membrane [32]. Extracellular release of eMI cargoes could represent an alternative way for cells to remove molecules particularly when degradation is impaired, and to execute immune signaling and pathogen surveillance. The fusion between the late endosome body and the plasma membrane is responsible for the formation of exosomes, a subset of extracellular vesicles enriched in endosomal proteins [33].

On the other hand, the fusion between late endosomes and lysosomes contributes to proteostasis and organelle turnover; moreover, it takes part in the autophagic response to aminoacid starvation. Indeed, it has been demonstrated that eMI activation can contribute in the case of prolonged starvation, acting as a regulatory pathway. Among its substrates, there are also some selective autophagy receptors (including p62 and LC3-II), probably degraded through the eMI pathway to avoid the activation of bulk macroautophagy [29]. Recent studies showed that eMI is preferentially activated by some selective stressors. They include high glucose conditions, H_2_O_2_ treatment, and genotoxic insults such as etoposide. On the contrary, serum starvation and short-time serum and aminoacid depletion treatment do not alter eMI activation. Notably, during oxidative stress, MAPK/JNK signaling is involved in eMI induction [32,34]. It has also been demonstrated a cross-talk between eMI and CMA as a compensatory mechanism. Indeed, when lysosomal-associated membrane protein 2A (LAMP2A) is knocked down and CMA blocked, eMI activity is increased; conversely, Vps4A/B knock-down blocked eMI and induced higher CMA activity [35].

### 2.2. Functions of Autophagy

The role of autophagy in cellular physiology is extensive, including adaptation to metabolic stress, degradation of potentially dangerous cargoes, and prevention of genomic modifications.

Glucose represents the major energy source for several cell types. In the case of low glucose levels, autophagy mediates the response aimed at meeting the intracellular energy requirements for cell survival through the catabolism of reserves and the recycling of intracellular macromolecules. A crucial hub responsible for glucose sensing is the highly conserved mTOR complex, composed of mTORC1 and mTORC2. Upon glucose availability, mTORC1 is activated to stimulate the anabolic process and promote proliferation [36]. Glucose scarcity inhibits mTORC1 both blocking cell growth and proliferation and inducing autophagy. Being the synthesis of macromolecules, mainly protein synthesis, the most expensive process in terms of energy, mTORC1-driven response blocks anabolism and promotes the usage of reserves.

However, beyond emphasizing the energy provided by autophagy, the energy required by autophagy itself has also to be highlighted. Thus, autophagy activation may depend on the ability of the cell to carry out the process spending the minimum amount of energy. Whether this ability is restricted, other vital processes are prioritized [37].

On the other hand, energy stresses responsible for autophagy induction also include nutrient excess, especially high glucose concentrations, resulting in an autophagy-mediated cell death, also known as “autosis”. Autosis is a programmed cell death, beyond apoptosis and necrosis, that is recognized by a large number of autolysosomes present in the cytoplasm and the fact that no proteins, aside from those belonging to the autophagic core, are required to mediate this type of death [38]. Indeed, autophagic cell death can be blocked by the depletion of ATG proteins [39]. In this condition, reactive oxygen species (ROS) have been identified as the major players in the overwhelmed induction of autophagy by activating the Mitogen-activated protein kinase (MAPK) signaling pathway [40]. Moreover, upon an overload of nutrients, ER stress can also occur following the increased protein entry in ER and the consequent augment of unfolded proteins. The unfolded protein response (UPR) is in turn responsible for activating autophagy [41]. Indeed, autophagy has an active role in protein turnover, orchestrating protein degradation together with the proteasome. The ubiquitin–proteasome system (UPS) is responsible for the degradation of about one-third of newly synthesized proteins. Even though UPS and autophagy are mechanistically different, an important common feature is the presence of ubiquitin sequence marking targets [42]. Generally, the majority of short-lived proteins are degraded by UPS, whereas long-lived proteins are preferentially degraded via autophagy, but this division is not strictly enforced. In addition, the relative contribution of autophagy depends on the cell type and the environment; indeed, this process contributes to cellular remodeling and adaptations to changes in the availability of different nutrients [43]. For instance, autophagy plays a pivotal role in physiological bone remodeling, which is crucial for the formation and maintenance of bone morphology and the repair of damaged bones. The differentiation of bone marrow mesenchymal stem cells requires AMP-activated protein kinase (AMPK)/mTOR signaling axis-mediated autophagy during the early stage. In contrast, protein kinase B (AKT)/mTOR signaling axis activation is needed in the late stage. The key role of autophagy in this differentiation process is also demonstrated by the loss of autophagic capability in fully differentiated osteoblasts. In this context, ROS are responsible for autophagy activation through MAPK induction [44]. Beyond bone marrow mesenchymal stem cells, it has also been demonstrated that autophagy is involved in the differentiation of several cell types, including immune cells [45], hematopoietic cells [46], neurons [47], by removing proteins that should not be present in each specific stage of differentiation.

The above-mentioned functions define autophagy as a crucial process to maintain homeostasis; consequently, perturbations of this degradation process are associated with numerous diseases, such as infections, cancer, and neurodegeneration [48].

Autophagy acts as an innate immune mechanism aimed at removing pathogens invading cells. This type of selective autophagy is known as xenophagy [49]. After the permeabilization of the phagosome through which the bacterium enters the cell, it will be promptly ubiquitinated and recognized by ubiquitin-binding autophagic receptors. Consistently, the deletion of genes codifying for key autophagic players determines an uncontrolled replication of bacteria in mammalian cells, making cells extremely susceptible to infection. For instance, murine myeloid-derived cells knock-out in the ATG5 gene show a higher vulnerability to *Mycobacterium tuberculosis* infection [50]. Knockdown of p62 or inactivation of ATG5 has been shown to increase viral capsid accumulation and accelerate cell death induced by the Sindbis virus [51].

Based on this, bacteria and viruses have developed mechanisms to elude autophagy degradation. In several cases, bacteria inhibit autophagy activation at different levels to carry on the infection or mask their surface to avoid ubiquitination [52]. For instance, *Francisella tularensis* escapes from autophagic destruction by coating its surface with polysaccharidic O-antigens that protect it against polyubiquitination [53].

Being autophagy a self-clearance pathway, among its functions there is also the removal of oxidized cellular components and, consequently, the regulation of intracellular ROS content. ROS are small molecules, including one-electron reduction products such as superoxide anions (O^•2−^), hydroxyl radicals (^•^OH), peroxyl (RO_2_^•^), and alkoxy (RO^•^), two-electron reduction products such as hydrogen peroxide (H_2_O_2_). ROS and RNS (reactive nitrogen species) are mainly produced in mitochondria by the electron transport chain, thus their production is strictly associated with the metabolic status of the cells. Basal levels of ROS have a physiological function of signaling, acting as second messengers, and activating transcriptional factors. At high concentrations, ROS/RNS can react with organelle and macromolecules resulting in their oxidation. The net content of ROS depends on the balance between ROS production and elimination, thanks to the action of antioxidant mechanisms [54]. Autophagy is part of the adaptive antioxidant response of cells, and it is responsible for the different capacities of cells to cope with ROS originating from different insults based also on a different ROS threshold required to activate autophagy [55].

## 3. Regulation of Autophagy

Autophagy is a homeostatic mechanism, and it is not surprising that autophagic levels can significantly fluctuate when cellular homeostasis is perturbed, regardless of whether it is the consequence of nutrient shortage, pathogen infections, or exposure to pro-oxidant agents. In all these cases, the autophagy rate is enhanced to cope with stress conditions and to preserve cell integrity and viability [56]. At the same time, it should also be considered that an excessive degradation of cell components can be detrimental to the cells and eventually lead to cell death [57]. The term autophagic cell death was introduced to identify a type of cell death that can be blocked by genetic inhibition of autophagy [58]. As high autophagic activity was reported in many dying cells and no specific morphological features of autophagic cell death have ever been identified, it is not trivial to discriminate between a cell death “by autophagy” and one that is only dependent or triggered by autophagy, like autosis [59]. Regardless of this difference, cells must fine-tune autophagy activity and duration to make sure that it is always proportional to the intensity of the stimulus to prevent undesired detrimental effects. A common trait of all types of autophagy is that they all require lysosomes to complete the process. Therefore, changes in lysosomal activity, positioning, and expression of lysosomal membrane proteins impact autophagy activity [60,61]. The regulation of the specific forms of autophagy, namely CMA and eMI, is still poorly studied but it generally occurs at either substrate recognition or at the lysosomal level [26,62]. On the contrary, the regulation of macroautophagy is well characterized and all steps of the autophagy pathway, from autophagosome formation and maturation to autophagosome fusion with lysosomes, are subject to regulation [3]. Regulation of macroautophagy can occur at multiple levels: transcriptional, post-transcriptional, and post-translational.

### 3.1. Transcriptional Regulation

The transcriptional regulation depends on the expression of autophagic genes; the increase of even a few of them is sufficient to stimulate autophagy. Several transcription factors have been shown to regulate autophagy, either activating or inhibiting it.

#### 3.1.1. TFEB

The transcription factor TFEB is considered the master regulator of autophagy and lysosomal biogenesis, and many genes involved in different steps of the autophagic pathway, including Beclin-1, LC3, and p62, are TFEB targets [63]. Consequently, the increase of TFEB expression causes activation of autophagy. Various transcriptional factors, in turn, can regulate TFEB expression, including CREB, which also regulates ATG7 and ULK1, by recruiting its coactivator CRTC2 [64]. TFEB can be also regulated through phosphorylation that sequesters it into the cytosol and prevents its nuclear activity. The main kinase responsible for TFEB phosphorylation in the presence of aminoacids is mTOR [65]. In particular, mTORC1 directly phosphorylates TFEB at Ser142 and Ser211 sites, inactivating it. 14-3-3 proteins also bind phosphorylated TFEB and prevent its nuclear translocation [63]. Moreover, AKT directly phosphorylates TFEB at Ser467 and represses its nuclear translocation independently of mTORC1 [66].

#### 3.1.2. FoxO

Beyond TFEB, members of the Forkhead Box O (FoxO) family can also regulate autophagic genes. FoxO3 induces a bulk induction of autophagy, determining atrophy in myotubes by promoting genes such as LC3 and GABARAPl1 [67]. Moreover, FoxO3 can induce autophagy in a FoxO1-dependent manner. Specifically, FoxO3 promotes the phosphorylation of FoxO1 by AKT1, causing translocation of FoxO1 to the cytoplasm, leading to the induction of autophagy [68].

#### 3.1.3. Peroxisome Proliferator–Activated Receptors (PPARs)

The transcription factors of the PPARs family, such as PPARα and PPARγ, regulate gene expression upon metabolic variations and have opposite roles in the regulation of autophagy. PPARα is activated by free fatty acids and it can bind promoters of autophagic-related genes, regulating the expression of LC3, BNIP3, ATG7, and Beclin1. Pharmacological activation of PPARα is sufficient to release autophagy inhibition in the fed state, inducing lipophagy, and PPARα knockout mice are partially defective in activating autophagy during fasting [69]. During adipogenesis, it has been demonstrated that PPARγ induces LC3, Beclin1, and ATG4b, directly regulating autophagy, and indirectly promotes the expression of TFEB and FoxO1 inducing autophagy to remove proteins not useful in the differentiation step ongoing [70].

#### 3.1.4. p53

The transcription factor and tumor suppressor p53 has a dual role in modulating autophagy, depending on its subcellular localization. Nuclear p53 activates ATG genes, among which ATG4A, ATG5, and WIPI1, and genes inhibiting mTOR, such as AMPK and TSC2. Another p53 target is DRAM1, which promotes the acidification of lysosomes and, consequently, autophagy. On the contrary, cytosolic p53 inhibits autophagy through different pathways, such as the inhibition of AMPK, mTOR activation, and the ubiquitination and degradation of Beclin1 [71].

#### 3.1.5. AMPK

A crucial stress sensor responsible for maintaining energy balance is AMPK, which also acts as an epigenetic regulator. Indeed, it can modulate the activity of histone acetyltransferases (HATs) and histone deacetylases (HDACs). Among HDACs influenced by AMPK, there is SIRT1, which induces autophagy by promoting the deacetylation of ATGs and FOXO1 genes. Under nutrient-deprived conditions, AMPK can phosphorylate GAPDH, which, in turn, translocates into the nucleus where it interacts with SIRT1 and activates its histone deacetylation function. This mechanism allows the release of epigenetic acetylation readers, such as bromodomain-containing protein 4 (BRD4), and the expression of autophagic and lysosomal genes. When nutrients are available, autophagy and lysosomal gene expression are repressed by the binding of BRD4 to the promoter and, consequently, the recruitment of a methyltransferase that induces histone demethylation [72]. Another target of AMPK is Acetyl-CoA synthetase short-chain family member 2 (ACSS2), whose function is to convert acetate into acetyl-CoA. When phosphorylated by AMPK, ACSS2 can translocate into the nucleus to interact with TFEB and to locally produce acetyl-CoA necessary for histone acetylation, inducing the expression of autophagic genes [73].

#### 3.1.6. HATs

The activity of acetyltransferase, including p300, can also be modulated by mTOR [72]. In particular, when activated, mTORC1 phosphorylates p300 and prevents the binding to the RING domain, inhibiting the catalytic function. As a consequence, autophagy induced by starvation is suppressed and lipogenesis is activated [74].

Regarding the epigenetic regulation of autophagy, DNA methyltransferases (DNMTs) and ten-eleven translocation (TET) proteins are responsible for methylation levels of several autophagic genes and, consequently, they can activate or suppress autophagy. For instance, TET1 can regulate the expression of ATG13 and DNA damage-regulated autophagy modulator protein 1 [75]; DNMT3 is responsible for methylating the DNA of the LC3 gene, resulting in a reduction of basal autophagy [76].

### 3.2. Post-Transcriptional Regulation

In addition to transcription factors, a significant number of non-coding RNAs (microRNA, circRNA, and lncRNA) participate in autophagy modulation. Among them, miR-101 inhibits RAB5A, ATG4C and, together with miR-376b, ATG4D; miR-103a-3p inhibits ATG5 [77].

### 3.3. Post-Translational Regulation

Post-translational modifications are widely present in autophagy regulation, comprehending ubiquitination, phosphorylation, glycosylation, methylation, acetylation, and protein lipidation (N-myristoylation, S-palmitoylation, S-prenylation, glycosylphosphatidylinositol (GPI) anchoring, and cholesterylation) [78]. Several of the abovementioned transcriptional factors are also responsible for directly regulating post-translational modifications of autophagic proteins, such as AKT, that phosphorylates Beclin1 at Ser295 and inhibits autophagy [79], whereas the phosphorylation at Ser91/94 by AMPK induces autophagy [80].

A crucial point of post-translational regulation of autophagy is the regulation of LC3 protein levels and function. The ubiquitin-activating enzyme UBA6 and the ubiquitin ligase BIRC6 cooperate and suppress autophagy by promoting LC3 monoubiquitination and proteasomal degradation [81]. LC3 phosphorylation at Ser12 by PKA suppresses its activity and inhibits autophagy. Metabolic and pathological inducers of autophagy caused dephosphorylation of endogenous LC3 [82].

## 4. Regulation of Autophagy by ROS

Oxidative stress is a major modulator of autophagy, as many stimuli that activate autophagy cause an increase in ROS. ROS can regulate autophagy both directly and indirectly. For instance, nutrient deprivation, the archetypal autophagy inducer, increases mitochondrial ROS levels, possibly due to an increased electron leakage [83].

A rapid accumulation of ROS during nutrient starvation promotes autophagy activation. Nutrient starvation stimulates the production of mitochondrial ROS, mainly H_2_O_2_, that can oxidize the cysteine protease ATG4, responsible for regulating LC3 interaction with autophagosomes. Oxidation inhibits ATG4 and promotes the lipidation of LC3 and autophagosome formation [84]. Since mitochondria are the main responsible for ROS production, ROS can promote mitophagy to avoid excessive oxidative stress. One of the mechanisms responsible for mitophagy activation by ROS is the induction of Parkin translocation to mitochondria, mainly driven by superoxide [85]. Additionally, the non-canonical mitophagic pathway regulated by BNIP3 can be modulated by ROS [86,87].

The regulation of autophagy by ROS can also be indirect, via modulation of autophagic gene expression; it is the case of Beclin1 and NF-kB expression prompted by ROS and that consequently activates autophagy [88]. Moreover, ROS can activate AMPK and stimulate autophagy to allow the cells to counteract oxidative stress and preserve cell viability. Several AMPK cysteine residues, like Cys299, Cys304, and Cys312, can be oxidized and modified, for instance by S-glutathionylation, causing a conformational change in AMPK that facilitates protein phosphorylation at Thr172. This event anticipates the activation of AMPK mediated by a high AMP:ATP ratio. [89].

ROS-mediated autophagy activation can also play a propaedeutic role in cell death by autosis. ROS-mediated loss of cardiolipin in neurons causes autosis [90], and high LPS-mediated activation induces ROS-mediated autophagic cell death in macrophages [91].

The ability of ROS to induce autophagic cell death can also be exploited as an anticancer strategy, as was shown in glioma [92], colon [93], and chronic myeloid leukemia [94]. ROS-mediated activation of autophagy can also trigger ferroptosis, an iron-dependent cell death mechanism, since autophagy regulates intracellular iron levels through ferritin degradation and transferrin receptor induction [95].

## 5. Mitogen-Activated Protein Kinases (MAPKs)

Mitogen-activated protein kinases (MAPKs) are a family of serine/threonine kinases that transduce extracellular and intracellular stimuli into a variety of cellular responses. The best-characterized members are the four conventional MAPKs: the extracellular signal-regulated kinases 1/2 (ERK1/2), c-Jun amino (N)-terminal kinases 1/2/3 (JNK1/2/3), p38 (p38α, β, γ, and δ), and ERK5 [96]. Other MAPKs have been described and classified as atypical MAPKs, because they are activated via different, often unclear mechanisms and their functions differ from those of conventional MAPK. Atypical MAPKs are ERK3/4/7/8 and Nemo-like kinase (NLK) [97,98].

Each conventional MAPK is the endpoint of a three-tier cascade starting with a MAP kinase kinase kinase (MAP3K) that is activated by upstream signals like cytokines or growth factors, often through G-protein-coupled or tyrosine kinase receptors [96].

**Figure 2:**
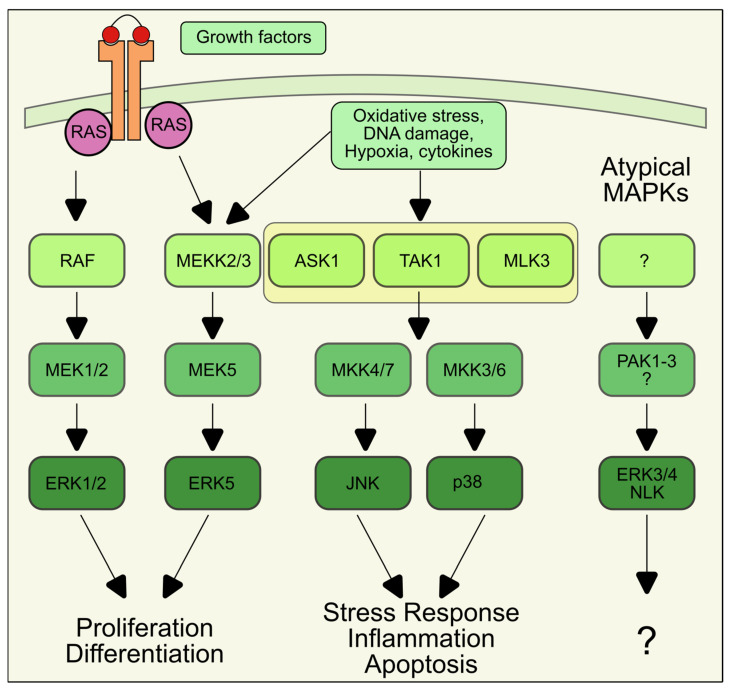
Cascades of conventional and atypical MAPKs. Conventional MARKs activate responses as proliferation, differentiation, and apoptosis whereas atypical MAPKs are involved in the autophagy response, but their roles are still unclear (?).

Active MAP3Ks phosphorylate and activate one or more MAP kinase kinases (MAP2Ks), which, in turn, phosphorylate-specific MAPKs on threonine and/or tyrosine residue located within a Thr-Xxx-Tyr motif [96]. MAPKs, in particular JNK and p38, can also be activated in response to several stressors originating from either outside or inside the cell, such as oxidative and genotoxic stress. An overview of the MAPK cascades and their output is shown in Figure 2. The activation mechanism of MAPK by oxidative stress is often not completely understood but it involves the activation of protein tyrosine kinase [99], inhibition of protein phosphatases [100], or direct activation of upstream kinases. One well-known example is represented by the MAP3K apoptosis signal-regulating kinase 1 (ASK1). ASK1 is usually inhibited by the binding of thioredoxin 1 (TRX1), a small antioxidant protein. Upon ROS production, TRX1 cysteine residues are oxidized, causing the release of ASK1 and its activation. Active ASK1 phosphorylates the MAP2Ks MKK3/4/6, which, in turn, activate JNK and p38 and induce the cell death pathway [101].

Once activated, MAPKs phosphorylate a considerable number of targets, either in the cytosol or in the nucleus, resulting in the modulation of a wide range of processes, such as cell survival, proliferation, differentiation, migration, and death [102]. In an over-simplistic view, the ERK pathway was traditionally considered associated with cell proliferation and differentiation, while JNK and p38 are involved in stress response and activation of cell death pathways. The presence of several isoforms of each MAPK with tissue specificity, the presence of scaffolding proteins and phosphatases that operate a spatiotemporal regulation of MAPK activation, and the connectivity among components of the MAPK pathway, add additional layers of complexity in the MAPK network and make the result of MAPK activation highly dependent on the type and duration of the stimulus and the cellular context [103].

## 6. Role of MAPK in the Regulation of Autophagy

**Figure 3:**
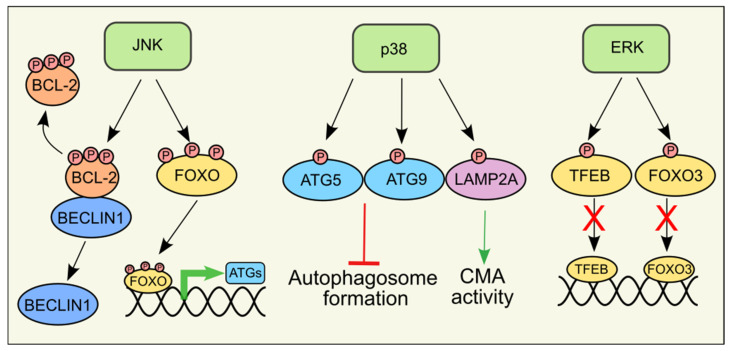
Regulation of autophagy by MAPKs. As downstream effectors of ROS, MAPKs can either activate (green arrows) or inhibit (red arrow with blunted end) autophagy by phosphorylating several downstream targets, promoting (black arrow), or inhibiting (black arrow with red crosses) their functions.

As downstream effectors of ROS, multiple reports have shown that MAPKs regulate autophagy, either activating or inhibiting it, depending on the stimulus and the cell type (Figure 3). However, the identity of the downstream effectors is not always known.

### 6.1. JNK

Several reports in the last decades have highlighted the role of JNK in the regulation of autophagy, specifically macroautophagy, and there is a consensus in defining JNK as a positive regulator. Mechanistically, JNK-dependent regulation of autophagy can be either transcriptional-independent or transcriptional-dependent. In 2006, Wei and colleagues demonstrated that JNK1, but not JNK2, phosphorylates the anti-apoptotic protein Bcl-2 upon nutrient deprivation [104]. The magnitude and/or the kinetic Bcl-2 phosphorylation by JNK1 determines whether Bcl-2 phosphorylation triggers a pro-survival (autophagy) or pro-death (apoptosis) response. Upon short-term nutrient deprivation, the phosphorylation by JNK1 at residues Thr69, Ser70, and Ser87 of Bcl-2 disrupts the binding with Beclin1, promoting autophagosome formation. In this condition, the interaction of Bcl-2 with the pro-apoptotic protein Bax is not affected, so the anti-antiapoptotic functions of Bcl-2 are preserved. If nutrient deprivation is prolonged, maximal Bcl-2 phosphorylation disrupts the binding with Bax, promoting apoptosis [105]. Thus far, this is the sole example of a direct involvement of JNK in the regulation of autophagy through direct phosphorylation of a protein linked to autophagy. The literature regarding the transcriptional-dependent regulation of autophagy by JNK is, on the contrary, quite rich. This is not surprising as many JNK downstream targets are transcription factors. JNK is a positive regulator of the FoxO transcription factors. Active JNK phosphorylates FoxO at several residues, promoting nuclear translocation and expression of its targets [106]. Several ATG genes, including LC3 and BNIP3, are among FoxO targets [107], and constitutive activation of FoxO3A was shown to be sufficient to induce autophagy [67]. In addition, FoxO can regulate autophagy through epigenetic mechanisms involving histone modifications that promote the expression of the autophagy and lysosomal biogenesis regulator TFEB [108]. Among the other JNK targets c-Jun, one of the subunits of the AP-1 transcription factor, upregulates Beclin1 expression to induce autophagy [109]. To date, nothing is known about the possible regulation of CMA by the JNK pathway, while a role in eMI has been reported in Drosophila. Here, the authors showed that JNK signaling mediates ROS-induced activation of eMI in the fly fat body, although the JNK targets are not yet identified [34].

### 6.2. p38

Similar to JNK, numerous reports are linking p38 activation to autophagy modulation. However, unlike JNK, there is conflicting data on whether p38 is a positive or negative regulator of autophagy. Regardless of the effect of p38 activation, regulation of autophagy seems to predominantly take place via phosphorylation of protein involved in autophagy. For example, ATG5, a protein involved in autophagosome formation, can be phosphorylated at Thr75 in a p38-dependent manner [110]. Phosphorylation of ATG5 by p38 blocks autophagy by inhibiting autophagosome maturation, highlighting a negative role of p38 on autophagy. In a similar paper, p38 was shown to inhibit autophagy in glial cells by phosphorylating ULK1 [111]. Additional reports demonstrated that p38 can inhibit autophagy also via indirect mechanisms. In 2010, the group of Sharon Tooze demonstrated that p38 inhibits autophagy by controlling the interaction of p38IP with ATG9, a member of autophagy core machinery [112]. Similarly, we showed that ROS-dependent activation of p38 indirectly limits starvation-induced autophagy via a feedback loop that decreases ROS levels by redirecting glucose metabolism from glycolysis to the pentose phosphate pathway (PPP), thus increasing NADPH production to preserve cell viability under conditions of nutrient deprivation [113].

While many papers indicate that p38 activation inhibits autophagy, others go in the opposite direction. Activation of p38 was shown to regulate autophagy through the phosphorylation of glycogen synthase kinase 3b (GSK3b) on Ser9 [114]. Whether p38 directly phosphorylates GSK3b was not clarified. More recently, p38 was shown to phosphorylate TFEB at Ser401, leading to TFEB activation to promote monocyte-to-macrophage differentiation [115]. In the paper, the effect on autophagy was not analyzed; however, being many autophagy and lysosomal genes among TFEB targets, it is reasonable to speculate that this activation would increase autophagy. The knowledge regarding the other types of autophagy is extremely limited. Only one paper highlighted the direct role of p38 in the regulation of CMA. The authors demonstrated that in response to ER stress p38 can localize to the lysosome and directly phosphorylate the CMA lysosomal receptor LAMP2A at Thr211 and Thr213, inducing LAMP2A oligomerization and CMA activation [116]. Another paper showed that p38 phosphorylates TFEB by inhibiting its activity. TFEB is a transcription factor promoting LAMP2 expression and, consequently, increasing activation of CMA. For this reason, several p38 inhibitors are used as CMA promoters since they induce TFEB translocation into the nucleus [117]. To date, nothing is known about whether p38 also influences eMI.

### 6.3. ERK1/2

The number of reports regarding the regulation of autophagy by ERK in conditions of oxidative stress is not huge, as it is more often studied in the context of the RAS/RAF/MEK/ERK pathway in tumors in which this pathway is altered. For instance, RAS or ERK blockade was shown to increase autophagy in pancreatic cancer [118]. Along the same line, ERK was shown to inhibit FoxO3, a positive regulator of autophagy [119], and to phosphorylate TFEB at Ser142 when sufficient nutrients are present, sequestering TFEB in the cytosol. In starvation conditions, ERK does not bind TFEB, leaving it to translocate into the nucleus [120].

Both lines of evidence agree on the inhibitory function of ERK on autophagy. Interestingly, ERK was shown to localize to the cytoplasmic face of autophagosomes and to interact with some ATG proteins. In this case, autophagosomes act as a scaffold in the spatio-temporal regulation of ERK phosphorylation in response to growth factors [121]. Whether active ERK localized at the autophagosome has any effect on ATG proteins and autophagy is not known. As for CMA and eMI, there are currently no reports on the regulation by ERK.

### 6.4. ERK5

A limited number of papers described the involvement of ERK5 signaling in autophagy, without an agreement on the output of its activation. The MEKK2/3-MEK5-ERK5 pathway was shown to promote basal mitophagy in cultured cells [122], while in neuronal cells MEK5 inhibition was shown to activate autophagy in a mTOR-independent and ERK5-independent manner [123]. Another paper indicated that ERK5 inhibition activates autophagy, but in this case, the effect was attributed to ER stress caused by ERK5 inhibition, rather than a direct modulation of ERK5 on autophagy [124].

### 6.5. Atypical MAPK

Very few reports investigated the influence of atypical MAPKs, in particular NLK, on autophagy. NLK was shown to be activated by stress conditions such as hyperosmotic and oxidative stress in HEK293 cells and to phosphorylate Raptor and inhibit mTORC1 [125], suggesting that NLK stimulates autophagy, although this possibility was not tested. A more recent paper by Tejwani and colleagues suggests an opposite effect of NLK. They show that in neuronal cells NLK inhibits lysosomal biogenesis and autophagy by promoting the destabilization of nuclear TFEB [126].

## 7. Conclusions

In detailing the fundamentals of the interplay between redox regulation and autophagy, this review is structured to provide an outline of the underlying principles of how MAPKs are contributing. For autophagy functioning cells use an orchestrated system, which includes multifaceted input cues that cause a signal to be generated. The general set-up is completed by appropriate feedback systems, the detailed mechanisms of which still need to be uncovered. Autophagy and MAPK are both modulated by several stimuli, and it is therefore not surprising that there is plenty of literature showing their interconnection, at least for what regards macroautophagy. The biochemistry of gene activation through redox-sensitive transcription factors has largely been elucidated. However, several key questions remain to be answered. One major question concerns the hierarchy of redox regulation. In this context, ROS are among the main stimuli responsible for the activation of autophagy through MAPKs. This makes cellular antioxidant defenses crucial because they regulate the amount of ROS imposing the threshold for autophagic activation [83]. As mentioned in the published literature and in several instances in the present review, there are the so-called master regulators, but the question arises: who regulates the regulator? Therefore, cell fate is regulated by cellular antioxidant capacity; in fact, cells with prominent antioxidant defenses, such as the aforementioned liver cells [127], cardiac cells [40], and bone marrow mesenchymal cells [44] exploit ROS to activate autophagy avoiding cell death. A better understanding will require network analysis in terms of feedback and feedforward loops at spatiotemporal patterns. It is not surprising that the final effect of MAPK activation is strongly cell-dependent and stimulus-dependent, reflecting the complex regulation of the MAPK pathways that determine their outputs. This depends on a better understanding of the molecular mechanisms linking MAPK and autophagy, as many papers are more focused on the final effects of MAPK modulation than on how the regulation takes place. Another important consideration concerns the duration of the stimulus, which determines whether the cellular response will be pro-survival or pro-death. In fact, the activation of autophagy is directly associated with the cell’s ability to survive under stress conditions, either by utilizing alternative energy sources or by engaging the autophagic pathway to eliminate the source of stress itself (e.g., misfolded proteins, bacteria, viruses). However, if the stimulus is prolonged, apoptosis tends to become the predominant outcome. This aspect further complicates the relationship between oxidative stress and the autophagic response, also introducing limitations in the potential therapeutic exploitation of this interplay. Similarly, a major research challenge exists in further elucidating the molecular details linking MAPK and other types of autophagy, whose regulatory mechanisms remain poorly understood.
